# Advances in the Potential Biomarkers of Epilepsy

**DOI:** 10.3389/fneur.2019.00685

**Published:** 2019-07-02

**Authors:** Dominik Kobylarek, Piotr Iwanowski, Zuzanna Lewandowska, Nattakarn Limphaibool, Sara Szafranek, Anita Labrzycka, Wojciech Kozubski

**Affiliations:** Department of Neurology, Poznan University of Medical Sciences, Poznan, Poland

**Keywords:** biomarkers, epilepsy, epileptogenesis, inflammation, neurological disorders, blood-brain barrier breakdown

## Abstract

Epilepsy is a group of chronic neurological disorders characterized by recurrent, spontaneous, and unpredictable seizures. It is one of the most common neurological disorders, affecting tens of millions of people worldwide. Comprehensive studies on epilepsy in recent decades have revealed the complexity of epileptogenesis, in which immunological processes, epigenetic modifications, and structural changes in neuronal tissues have been identified as playing a crucial role. This review discusses the recent advances in the biomarkers of epilepsy. We evaluate the possible molecular background underlying the clinical changes observed in recent studies, focusing on therapeutic investigations, and the evidence of their safety and efficacy in the human population. This article reviews the pathophysiology of epilepsy, including recent reports on the effects of oxidative stress and hypoxia, and focuses on specific biomarkers and their clinical implications, along with further perspectives in epilepsy research.

## Introduction

Epilepsy, a condition affecting the central nervous system (CNS), is characterized by the occurrence of repeated seizures along with a chronic complex of somatic, vegetative, and psychiatric symptoms. Epilepsy can be defined as when the patient experiences at least one of the following: (a) two or more unprovoked (or reflex) seizures more than 24 h apart, (b) one unprovoked (or reflex) seizure and, over the next 10 years, a recurrence risk of at least the general recurrence risk (60%) after two unprovoked seizures or (c) a diagnosis of an epilepsy syndrome. Patients with epilepsy are prone to generate epileptic seizures and consequential social, psychological, cognitive, and neurobiological disabilities (1). It is estimated that 1–2% of the world's population is affected by epilepsy (2, 3). It may occur in all age groups and is connected with a burden of socioeconomical, behavioral, psychiatric, and other medical issues for both the patient and their close ones (1, 4).

Epileptogenesis describes the process of structural modifications leading to seizure activity in a normal brain (5). Throughout recent years, many hypotheses have been proposed to explain the underlying etiopathogenesis of epilepsy, including neurodegeneration (6, 7), disturbance of brain-blood barrier (BBB) (8), amygdala dysregulation, alterations of the glutamatergic system (9), oxidative stress (10), hypoxia (11), and the epigenetic modification of DNA (12). Moreover, the majority of studies on inflammation and epilepsy indicate the important role of inflammatory markers in epileptogenesis through the dysregulation of cytokine balance in the CNS or through the complement pathway. These hypotheses may not exclude one another and may in fact be concurrently presented leading to the culmination of epilepsy. As ~40% of cases of epilepsy have an unknown etiology, further investigations into the potential causes are essential in order for physicians to provide an optimal treatment for patients (13).

WHO defines biomarkers as “almost any measurement reflecting an interaction between a biological system and a potential hazard, which may be chemical, physical, or biological. The measured response may be functional and physiological, biochemical at the cellular level, or a molecular interaction” (14). The role of finding novel markers in post-epileptic brain damage is a possible grasping point for the prevention of complications and for the development of targeted methods of treatment in the future. The need of an investigation into new biomarkers is also augmented by the relatively low specificity of EEG, which remains the main diagnostic tool in epilepsy (15). Biomarkers may play a role in individualized epileptic treatment, based on the patients' biomarker profile. As there are many types of epileptic conditions, each condition would have a certain panel of biomarkers. Biomarkers would also play a role in monitoring anti-epileptic treatment and may have a potential value in determining patients who would benefit more from surgical therapy.

## Neuroinflammation and Oxidative Stress

Neuroinflammation is considered to be a primary factor in epileptogenesis. Reactive oxygen species (ROS) has been indicated to play a crucial role as mediators in the process of neuronal injury (16–18). Currently, there are two suggested pathways of ROS production, the non-enzymatic and the enzymatic pathway. The non-enzymatic pathway is indicated to be triggered by the ionization process, UV radiation and toxic influence of chemicals and drugs. The enzymatic pathway, on the other hand, is a result of intracellular damage by enzyme-mediated processes including respiratory chain, xanthine oxidoreductase (XOR), peroxisomal oxidases, enzymes of the cytochrome P450 family, cyclooxygenases (COX), lipoxygenases and NADPH oxidases (NOX). ROS is considered to be a waste product of these enzyme-mediated reactions (19). Recent studies suggest that ROS may play a crucial pro-epileptic role including pro-inflammatory cytokine production and microglial activation during epilepsy. McElroy et al. have additionally proposed the role of ROS in modulating the course of neuroinfection (20).

The increased production of ROS leads to microglial activation, ultimately resulting in the release of pro-inflammatory cytokines (20). Cytokines play an essential role in these processes not only because they are responsible for the aggravation of immune response, but also because they regulate the pro- and anticonvulsive neuronal hyperexcitability (21, 22). In light of McElroy et al.'s investigations, this concept was supported by the results of decreased microglial activation through redox-sensitive m-Tor pathway following the administration of anti-oxidative factors (20).

Interestingly, other studies have demonstrated that the main cytokine activator, cyclooxygenase-2 (COX-2), was triggered via ROS through transforming growth factor-B-activated kinase 1 (TAK1) pathway (23, 24). These investigations brought together ROS, COX-2 TAK1 pathway in the neuroinflammatory process. To support this concept, it was verified that the temporal lobe epilepsy (TLE) is associated with microglia activation, which in turn leads to the production of ROS and other cytotoxic factors (25–28). The activation of microglia through oxidative stress promotes the apoptosis of pericytes through ROS elevation (29).

### The Role of HMGB1 in Oxidative Stress

High mobility group box-1 (HMGB1) has recently emerged as a potential biomarker of epilepsy (30). It takes part in the immune response via activating macrophages and endothelial cells, leading to the release of tumor necrosis factor-a (TNF-a), interleukin-1 (IL-1), interleukin-6 (IL-6) by connecting to the receptor for advanced glycation end products (RAGE) and to TL4 (Toll-like receptor 4). This specific connection triggers NF-kB (nuclear factor kappa-light-chain-enhancer of activated B cells) activation and thus the elevation of pro-inflammatory proteins levels (31). Furthermore, via stimulating TLR4 and neutrophils, HMGB1 is the factor that leads to oxidative stress (32, 33). The HMGB1-mediated HMGB1-TLR2/4-NF-κB pathway has been shown to take part in epileptogenesis via microglial activation. HMGB1 has been additionally indicated as a potential therapeutic agent in epilepsy and as a non-invasive biomarker, which could identify patients with high risk of epilepsy (34). The level of HMGB1 has been shown to increase within 3–4 h after a drug-resistant epilepsy (DRE) seizure, proving HMGB1 to be a promising marker (35). Zhu et al. also reported the elevation of HMGB1 within 24 h after an episode of seizure in children, in comparison to the control group. The authors suggested that HMGB1 can be a prognostic factor of the frequency of seizures in the course of epilepsy (36).

### Hypoxia and Epilepsy

**Figure 1:**
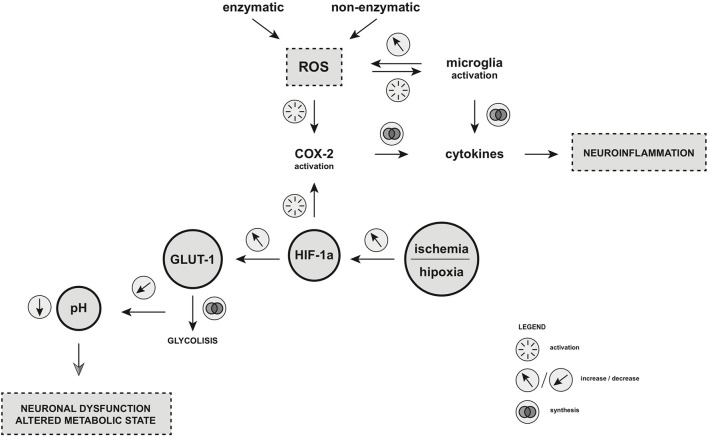
Oxidative stress and hypoxia as the key players of epileptogenesis. Enzymatic and non-enzymatic pathway of ROS production as well as increased level of HIF-1α under hypoxic/ischemic condition leads to COX-2 activation. As the result, microglia is activated and cytokines production is augmented, which leads to neuroinflammation. HIF-1α is also the factor that regulates the glucose metabolism in the central nervous system (CNS) through GLUT-1 synthesis. Dysregulation of HIF-1a production may result in an accumulation of pyruvate in neuronal cells which is then converted into butyric acid via butyric dehydrogenase. This accumulation of by-products may lead to a decreased pH within the inner environment of neuron, leading to its dysfunction and altered metabolic state. ROS, reactive oxygen species; COX-2, cyclooxygenase-2; HIF-1α, hypoxia inducible factor-1 alpha; GLUT-1, glucose transporter-1. Illustration by Paulina Szuba.

Hypoxia resulting from ischemic events can lead to the energetic disturbances of homeostasis. The following dysregulation of ATP-dependent ion-pumps drives the imbalance of sodium, calcium, and potassium ions concentration, leading to the release of excitatory amino-acids such as glutamate (37, 38). As a consequence, an uncontrolled electric stimulation is provoked, resulting in cellular brain injury (39–41). Hypoxia inducible factor (HIF-1), a heterodimer protein consisting of two subunits a and b, is involved in ischemic processes (6). The level of HIF-1a depends on the partial concentration of oxygen (42). In normoxemia, HIF-1a is rapidly brought down by the protein von Hippel Lindau (pVHL)-mediated process of ubiquitin-proteasome pathway. Hypoxia, on the other hand, blocks the degradation of HIF-1a, resulting in its accumulation within the cell (43). Factors responsible for the stabilization of HIF-1a may include insulin, insulin-like growth factor, platelet-derived growth factor (PDGF), epithelial growth factor, and interleukin-1B (41). Moreover, HIF-1a is an important regulator of gene expression in the peripheral tissues and the CNS which is currently in a hypoxic state. The effect of HIF-1a is the promotion of physiological processes including angiogenesis, glycolysis and glucose transporter 1 (GLUT1) membrane recruitment (44). The final effect of glycolysis is the accumulation of pyruvate in neuronal cells which is then converted into butyric acid via butyric dehydrogenase. This accumulation of by-products may lead to a decreased pH within the inner environment of neuron, leading to its dysfunction and altered metabolic state. The ketogenic diet which is based on lowered glucose intake may omit this pathway regulated by HIF-1a and alternatively promote beta-oxidation, converting substrates to acyl-CoA (45). Taking this into consideration, the efficacy of a ketogenic diet for patients with DRE can be beneficial. In addition, it has been shown that a ketogenic diet also improves the outcomes of patients with GLUT-1 deficiency syndrome (46). Studies on epilepsy-induced rat models and post-mortem human histopathologic brain samples supported a significant correlation between HIF-1a elevation and epilepsy occurrence (42–44, 47) Numerous analyses have supported the positive correlation between HIF-1 and the elevation of COX-2 production. It has been demonstrated that HIF-1a binds to hypoxia responsive element on the COX-2 promotor located in DNA, resulting in the up-regulation of COX-2 and PGE-2 (prostaglandin E2) (48). This could potentially explain the mechanism of febrile seizures in pediatric patients, seizures resulting from perinatal ischemia, and seizures occurring after strokes and transient ischemic attacks (TIA). Investigations and further understanding of the basis of hypoxia along with oxidative stress as the underlying cause of epilepsy could lead to the discovery of new potential epilepsy biomarkers. For the first time, we suggest that both pathways of hypoxia and oxidative stress may contribute to brain damage and epileptogenesis through COX-2 activation (Figure 1).

## MicroRNA as the Novel Diagnostic Tools for Epilepsy

MicroRNAs (miRNAs) are a group of single-stranded, endogenous, non-coding molecules. It is estimated that 1–5% of both animal and human genes are involved in the coding miRNA (49). To date, over 500 genes which take part in miRNA coding have been discovered and the number is still rising. miRNAs take part in both physiological and pathological processes through its regulation of homeostasis. Research has revealed the involvement of miRNAs in cellular processes including cellular division, cellular cycle control, cell differentiation, apoptosis, angiogenesis, and oncogenesis (50).

Moreover, miRNA is involved in the immunological system through its regulation of the immune responses during infection (51–54). For this reason, miRNAs have been suggested to be involved in epileptogenesis (55). Three hypotheses explaining the origin of miRNA in biofluids were proposed. The first hypothesis suggests the passive entry of miRNA into the systemic circulation as a result of mechanical cellular damage, which may take place during neuroinflammation. The second hypothesis presumes that miRNAs enter the circulation via the actively-secreted microvesicles (MV), which could also be involved in intracellular communication. The third hypothesis proposes that miRNAs may be actively secreted as a response to a large variety of stimuli. This mechanism is preceded by the formation of the complex of miRNA-Argonaut proteins (Ago) and HDL (56, 57).

Due to the feasibility of comparing histopathological nervous tissue samples from both human and animals, the biological processes involving miRNAs have been extensively studied. It has been suggested that epigenetic modifications implicated in the development of DRE through the modulation of gene expression are involved in the absorption of anti-epileptic drugs (58).

Due to miRNA profiling in patients with epilepsy, the significance of miRNA in the regulation of protein levels in epileptogenesis has been identified. Elevated levels of miRNA-23a, miRNA-34a, miRNA-132, miRNA-146a in epilepsy, in particular, were frequently detected. Additionally, elevated levels of miRNA-21, miRNA-29a, miRNA-132, identified as regulated by p53, were noticed subsequent to episodes of seizures (59). The significance in the plasma levels of miRNA-134 within the course of antiepileptic drugs usage has been reported, in which miRNA-134 could potentially serve as a peripheral biomarker reflecting the acute epileptic episode during the course of the treatment (60). Similarly, miRNA-4521, as reported by Wang et al. (61), could serve as a potential biomarker in refractory epilepsy. It has also been stated that the levels of miRNA-301a-3p collected from the blood were different in patients with DRE compared to epileptic patients who were responsive to therapy. Thus, it was suggested that miRNA-301a-3p could be a marker for an early diagnosis of DRE (59). In another study, the silencing expression of miRNA-132 was shown to lead to a decreased number of seizures. It was suggested that the silencing of miRNA-132 has an impact on the MFs-CA3 pathway, which may provide beneficial outcomes for patients with epilepsy (62).

Table 1 presents the recent reports on miRNA detected in biofluids from patients with epilepsy.

**Table 1:** Reports on miRNA detected in biofluids from patients with epilepsy.

**miRNA**	**Regulation**	**Species**	**Material**	**Comments**	**References**
hsa^*^-miR-30a-5p	Up-regulation	Human	Biofluid	Expression was analyzed by microarray and RT-qPCR. MiR-30a was overexpressed in the serum of epilepsy patients during seizures onset. The expression of miR-30a was positively associated with seizure frequency.	(63)
mir-143-3p; mir-145-5p; mir-365a-3p; mir-532-5p	Up-regulation	Human	Biofluid	Up-regulated in serum in patients with mTLE. MiRNA measured 30 min after seizures	(64)
miR-106b; miR-146a; miR-301a	Up-regulation	Human	Biofluid	Up-regulated levels in serum derived from patients with epilepsy in comparison to healthy control group	(65)
miRNA-129-2-3p	Up-regulation	Human	Biofluid	Upregulated miR-129-2-3p confirmed by qRT-PCR expression in plasma samples of refractory TLE group	(66)
hsa-miR-342-5p; hsa-miR-4446-3p; hsa-miR-30b-5p	Down-regulation	Human	Biofluid	Downregulated in DRE group compared to drug-responsive group and control group	(67)
hsa-miR-134-5p	Down-Regulation	Human	Biofluid	Downregulated in plasma samples from MTLE patients when compared with healthy controls	(68)
hsa-miR-194-5p; hsa-miR-15a-5p; hsa-miR-144-5p; hsa-miR-181c-5p; hsa-miR-889-3p	Down-regulation	Human	Biofluid	Downregulated in serum in patients with epilepsy	(67)
hsa-let-7d-5p; hsa-miR-106b-5p; hsa-miR-146a-5p; hsa-miR-130a-3p	Up-regulation	Human	Biofluid	Upregulated in serum samples from TLE patients	(59)

* *hsa, homo sapiens*.

### The Role of miRNA in the Brain Blood Barrier Damage

Elevated levels of miRNA-132 were observed in animal models of CA3 status epilepticus (SE). Microinjection of antagomir against miRNA-132 in animal models have additionally been found to produce an anti-inflammatory effect (62). As the concept of inflammation and BBB damage has been proposed, the link between miRNA-132 and epileptogenesis was indicated (69). Elevated levels of miRNA-34a after SE has additionally been supported. Following the microinjection of antagomir against mir-34a, an inhibition of caspase-3 was reported, suggesting a possible association with increased neuronal survival and decreased level of nerve tissue apoptosis. In turn, studies on animal models and people with TLE have demonstrated the regulatory role of miRNA-146 during epilepsy (70, 71).

### Oxidative Stress and miRNA

Increasing number of studies indicate a role of oxidative stress as the underlying cause of many diseases. Cellular redox signals are mediated by miRNA, an important regulator of homeostasis. On the basis of epigenetic modification, miRNA regulates ROS at the stage of post-transcriptional degradation of NOX4 and Nrf2rna, which is the down-regulatory mechanism of ROS production, resulting in decreased synthesis of ROS (72, 73). For example, miRNA-129-5p negatively regulates HMGB1 during epilepsy. The TLR4/NF-kB signaling pathway is activated by elevated levels of HMGB1. It has been shown that miRNA-129-5p plays a role in the inhibition of the development of autoimmune encephalomyelitis-related epilepsy rat model by targeting HMGB1. Dysregulation of miRNAs' physiology involved in maintaining ROS homeostasis may possibly lead to oxidative damage and disease progression (74).

### Hypoxia and miRNA

Under conditions of hypoxia, the expression of HIF1A mRNA is elevated and HIF-1a protein stabilization is increased. HIF1, the intracellular messenger of hypoxia, is transferred to the nucleus and regulates the expression of target genes. HIF1 binds to the HRE sequence to the cluster mir-200a-mir429 on chromosome 1, leading to an increased expression of miRNA-429. Subsequently, miRNA-429 in the cytosol binds to a sequence located in the 3'UTR of the HIF-1a miRNA, leading to the decreased activity of HIF-1 (75). The upregulation of miRNA-429 in human hippocampal tissues from TLE and hippocampal sclerosis-convergence have been further supported, indicating the high utility of this miRNA (76).

### Circulating miRNA as Biomarker: Prospects and Limitations

For decades, circulating miRNAs have been a research material of interest. In contrast to the miRNA samples obtained from invasive surgical procedures, biofluids, particularly blood-derived plasma and serum, is easily accessible. A full blood or serum test is minimally invasive compared to procedures such as a lumbar puncture. miRNA studies in biofluids have become increasingly accessible and applicable due to the development of new molecular investigative methods. The possibility of using miRNA derived from blood as a sensitive marker, both as a prognostic and predictive factor of many diseases, is invaluable for modern researches (77–79).

On the other hand, many reports point to the uncertain efficacy of circulating miRNA. The origin of miRNA in the bloodstream remains unclear. In addition, reports point to the equivocal specificity of miRNA, which can be modified under the influence of various extrinsic factors such as tobacco, pregnancy, diet, or alterations to the circadian cycle (80–82).

## The Role of the Complement System in Epilepsy

The complement system is composed of more than 30 proteins which interact in a strictly organized manner to destroy pathogenic agents and to protect normal tissues from the deposition of immune complexes (83). There are three pathways leading to complement activation: classic, alternative and lectin (84). Each pathway leads to the activation of fragment C3, which is cleaved to form opsonin C3b and C3a, promoting the activation of the lytic pathway, acting as anaphylotoxin and causing damage to cell membranes and pathogens. C5a formed through this process attracts macrophages and neutrophils, and also activates mast cells (85).

The complement system plays a critical role in the innate immune system and is one of the main mechanisms of the effector adaptive humoral response (86). It mediates the reaction against infectious agents through a coordinated sequence of the enzymatic cascade, leading to the elimination of foreign cells by pathogen recognition, opsonization, and lysis (87). Although it is essential in maintaining immune balance, inappropriate activation of the complement cascade can lead to tissue damage and contribute to the development and progression of various pathologies (88).

### Increased Concentrations of Ingredients, Biomarkers

Studies from human and animal models have indicated that the regulation of the complement cascade contributes to the development of epilepsy (89). The concentration of serum C3 in untreated patients with epilepsy were shown to be significantly higher than in that of healthy controls (90). Recent studies have reported an elevated concentration of the classical pathway components in patients with epilepsy compared to healthy controls and in untreated epileptic patients compared to those who are undergoing treatment (91–94).

Investigation into the plasma concentrations of a panel of complement analytes in epileptic subjects presented a highly predictive model comprising of 6 complement analytes (C3, C4, properdin, FH, C1Inh, and Clu) which distinguish between epilepsy cases and controls (89). This may useful for the development of prognostic markers and effective epilepsy therapies.

### The Classical Pathway

In the classical complement pathway, the proteolytic cleavage of the C3 fragment into C3a and C3b requires the linkage of the C1q to cell surfaces, C1s and C1s proteases (95).

Soluble C3a promotes the recruitment of microglia and inflammation, whereas C3b can be subsequently split into C3bα, C3bβ, and iC3b, all of which can act as opsonins. Recent studies have shown new non-canonical roles for phagocytic C1q-C3 signals in improving synaptic connectivity (96, 97). For instance, the C1q and C3b analytes are associated with the removal of synapses during the development of the visual system and the elimination of unwanted structures of synaptic hippocampus in models of neurodegenerative disorders (98–101). This indicates the role of the classical complement pathway in the epileptogenic remodeling of synaptic circuits associated with status epilepticus and TLE (92, 102–104).

Furthermore, it has been shown that the C1q-C3 signaling can modify the expression of the pro-inflammatory tumor necrosis factor alpha (TNF-α) and interleukin 1 beta (IL1β) (105, 106). In turn, the upregulation of TNF-α levels in microglia has been observed in the condition of the SE-induced activation of C5 (107). It has been further indicated that the SE-provoked increases in C1q signaling and the generation of C3a and C3b-mediated activation of C5a/b may contribute to the initiation and/or preservation of neuroinflammation in epilepsy (108). Further investigation would be required, however, to deepen the understanding of complement cascade in this matter.

The C1q analyte has been additionally proven to prevent further necrosis and inflammation by promoting phagocytosis of cellular debris and apoptotic cells (109), which could be considered a neuroprotective mechanism subsequent to SE (110, 111). C1q has also been shown to reduce the lipopolysaccharide-induced microglial release of IL-6 and TNF-α, and thus may play a role in helping to reduce the pro-inflammatory responses induced by SE (105, 112). Regardless of pathway activation, the final stage of the complement cascade leads to the formation of a membrane-attacking complex (MAC). MAC joins the cell membranes, creating a porous functional channel, which leads to the flow of ions and ultimately to the osmotic lysis of the attacked cell (113). The infusion of single proteins of the membrane attack the complex pathway (C5b6, C7, C8, and C9) to the hippocampus of awake, freely moving rats has been shown to induce cytotoxicity and behavioral and electrographic convulsions (83).

### Therapeutic Potential

The therapeutic implications in modulating the complement cascade has been previously demonstrated (114). The anti-C5 antibody directed toward the final complement pathway is of high therapeutic significance^.^ Treatment with eculizumab blocks the cleavage of C5 and prevents the formation of MAC while leaving the rest of the complement system intact (115). Most importantly, eculizumab appears to be well-tolerated in all approved clinical settings (116). Eculizumab and other designed inhibitors of the complement cascade are likely to achieve clinical utility that goes far beyond paroxysmal nocturnal hemoglobinuria and atypical hemolytic uremic syndrome, including autoimmune disease, transplantation, neurodegenerative, and other CNS diseases, including epilepsy (117, 118).

## Role of Cytokines in Epileptogenesis

Recent clinical and experimental findings have supported the premise of inflammation as a major pathological basis in epileptogenesis. Inflammation can be studied through the measurements of inflammatory cytokines, which are soluble mediators of cell communication that are critical in immune regulation. Inflammatory cytokines' potentiation of free radical species and alterations in glutamatergic neurotransmission, ultimately result in neuronal excitoxicity, and consequential structural alterations (such as BBB disruption) within the brain which have been consistently observed in epileptic individuals (119, 120).

Within the CNS, cytokines are produced as a response to various inflammatory stimuli. In recent years, studies have shown that epileptic seizures can induce the production of cytokines, which in turn contributes to further inflammation and structural changes, thereby establishing an ongoing cycle of events contributing to the development and progression of epilepsy (121). Both pro- and anti-convulsive effects have been reported for cytokines, suggesting the diverse nature of cytokine networks and the complex relationship between the immune system and epilepsy. Here, we review the different mechanism of cytokine involvement in the development of epilepsy.

### Free Radical Generation

Pro-inflammatory cytokines are indicated to inhibit neurogenesis through the direct induction of neuronal death via reactive oxygen species (ROS) generation and excitotoxic mechanisms. Due to its high intrinsic metabolic rate and low levels of protective antioxidants, the brain is highly susceptible to free radical neuronal damage. The generation of ROS from a preceding inflammatory event may result in progressive oxidative damage, cellular destruction and neuroprogression (122). Pro-inflammatory cytokines including IL-1B, TNF-α, and IFN-y have been shown to potentiate the effects of these free radicals (123). Consequentially, mechanisms of neuroprogression, including neurodegeneration and reduced neurogenesis, play a part in the underlying pathophysiology of the epileptic brain.

### Alterations in Glutamatergic Neurotransmission

Alterations in glutamatergic neurotransmission could trigger neuronal excitotoxicity, impaired neuroprotection, and the necessary conditions for the development of epilepsy (124, 125).

IL-1B has been indicated to alter the glutamate transporter expression leading to a decreased reuptake of glutamate. Resulting excess synaptic glutamate lead to subsequent N-Methyl-D-aspartic acid *(*NMDA)-mediated excitotoxicity and cellular damage (126). Particularly within the neurons of the hippocampus, the binding of IL-1B to the IL-1 receptor induces the phosphorylation of the NMDA receptor and the potentiation of its activity. This results in an increased neuronal calcium influx and subsequent cell death (127).

### Blood-Brain Barrier Compromise

Neuroinflammation induces structural changes to the brain parenchyma, one of which is the leakage of the BBB and thereby the changes in its functional properties (21). These alterations lead to cellular damage and neuronal hyperexcitability, leading to the reduced threshold for seizure induction. BBB disruption can be triggered by direct insult to the endothelium or via systemic factors, including activation of circulating leukocyte and release of molecular mediators that increase vascular permeability (128). Studies have shown BBB failure after exogenous administration of pro-inflammatory cytokines including IL-1, IL-6, TNF-a, and IFN-y, suggesting a link between the systemic immune system and neuronal dysfunction (129).

### COX-1 and COX-2

COX-2 is indicated to play an important role in the post-seizure inflammation and hyperexcitability of the brain, possibly contributing to secondary damage in the brain and the increased likelihood of repetitive seizures. One pathway is through their synthesis of PGE2, which has excitatory effects. Activation of a single PGE2 receptor (EP2) has been shown to exacerbate the rapid upregulation of IL-6 and IL-1B in activated microglia and reduce the production of TNF-a, IL-10. EP2 thus regulates innate immunity in the CNS by alternating the balance between pro- and anti-inflammatory cytokines (130).

Particularly in DRE, the cellular expression of COX-1 and COX-2 and relationship to the efflux transporter expression is particularly important for elucidating the underling effects of inflammation. The “transporter hypothesis” of DRE suggests that the overexpression of ATP-binding cassette (ABC) transporters such as P-glycoprotein (p-gp) and BCRP at BBB may prevent anti-epileptic drugs from reaching their targets. P-gp, an ATP-dependent efflux pump, has the function of pumping foreign substances out of the cell. P-gp up-regulation was in part caused by elevated COX-2 activity and pharmacologic inhibition of COX-2 has been shown to allow greater uptake of P-gp substrate phenytoin (131, 132).

The contribution of COX-2 inhibition in neuroprotection and its potential role in adjunct therapeutic strategy remains inconclusive, as there has yet to be a selective COX-2 inhibitor which has shown a favorable therapeutic outcome. Although short-term exposure might be useful, the accompanying risk of cardiovascular adverse effects makes it unlikely that chronic COX-2 inhibition can be used in the long-term treatment of epilepsy (130).

### Pro-Inflammatory Cytokines in Epilepsy

The seizure-induced activation of the cytokine network may suggest the interplay of the nervous-immune-endocrine systems in the pathological process of epileptic seizures. IL-1B and IL-8 are pro-inflammatory cytokines that activate additional cytokine cascades and increase seizure susceptibility and organ damage, whereas IL-1 receptor antagonist and IL-10 act as anti-inflammatory cytokines that have protective and anticonvulsant effects (22). It remains unclear whether increased cytokine levels in plasma and CSF of epilepsy patients relate to a cerebral inflammatory process alone, or arise as a result of postictal peripheral muscular recovery or circulating immune cells. Several studies related cytokines to changes in neuronal excitability and suggested a potential role for targeted therapy (21, 133).

#### IL-1B

While IL-1 cytokines are constitutively expressed at very low levels in the human CNS, they are often elevated in the brain under certain pathological states such as during an active seizure, hypoxic injury, and during the process of an infection (22). Recent clinical studies have reported changes in levels of IL-1B in the blood, CSF, and brain tissue (22). A significant difference was found where the level of in IL-B in CSF was increased in patients with generalized tonic-clonic seizures compared to the control group. The increased levels also show a significant positive correlation with the duration and frequency of seizures (134). A decrease in IL-1ra/IL-1B ratio was reported after a seizure, that leads to increased influence of the pro-inflammatory IL-1B and may implicate a pro-inflammatory state in the brain (135).

Other studies found no significant differences in the IL-1B concentration in blood and CSF after generalized tonic-clonic seizures (22). Studies in patients with focal epilepsy, mesial TLE, and febrile seizures similarly showed that postictal plasma concentrations of IL-1B did not significantly differ from baseline levels (21, 127, 136, 137). However, an increased level of IL-1B was found in post-mortem samples of patients with TLE when compared to autopsy controls (127).

Therapies for auto-inflammation including IL-1 blockade have been indicated in the treatment of refractory epilepsy (138). Febrile infection-related epilepsy syndrome, a rare but devastating encephalopathy occurring after a febrile illness, showed an improvement with anakinra while in refractory status epilepticus. This suggests that this treatment may be a useful adjunctive medication for certain cases of refractory epilepsy syndromes (139).

#### IL-6

IL-6 is a primary pro-inflammatory cytokine involved in the acute phase of the immune response. Seizures cause changes in levels of IL-6 both in CSF and in the peripheral blood. The magnitude of these changes is related to the severity of seizures. IL-6 levels are strongly increased after recurrent GTCS, whereas after single tonic-clonic or prolonged partial seizures IL-6 levels are increased to a lesser extent (140, 141). IL-6 levels have been reported to be significantly higher in the daily generalized motor seizures than in either intermittent seizures or in control subjects (142). This indicates the positive correlation between the magnitude of IL-6 activation and severity of cerebral epileptic activity. A meta-analysis of serum IL-6 levels in TLE patients revealed marginal but significant IL-6 elevation when compared to controls (143). While IL-6 seems to be consistently increased in epilepsy patients, it is not possible to explain whether it is a cause or consequence of the disease. A case study indicated that after blocking IL-6R with the monoclonal antibody tocilizumab, stable remission of epileptic symptoms could be achieved. This suggests the possible therapeutic implications and efficacy of tocilizumab in the treatment of synaptic diseases which needs to be further confirmed by controlled studies (144).

#### IL-8

IL-8, a pro-inflammatory cytokine, plays a role in the promotion of neuronal growth after injury and in the stimulation of nerve growth factor production, constituting both damaging and reparative functions involved in the pathogenesis traumatic brain injury. IL-8 is found to be significantly increased in the serum of patients with partial onset seizures (145). It is reported to be associated with seizure severity (measured by seizure frequency, VA score, or NHS3) in TLE, extra-temporal lobe epilepsy, and idiopathic generalized epilepsy (136). In neonatal seizures, IL-8 levels significantly increased within 24 h and remained increased after 48 and 72 h (22).

#### TNF-a

Although TNF-a is a prominent pro-inflammatory marker, there are limited reports on the significance of TNF-a in epileptic patients. No significant differences were found in the serum of patients with daily or intermittent generalized motor seizures (142). A study reported decreased frequency of CD8+ T-lymphocytes expressing TNF-a in mTLE patients, in which lower frequency could be explained by the migration of pro-inflammatory CD8 T-cells to brain areas affected by repetitive seizures, thus reducing their frequency in the peripheral blood (133). A study reported that in DRE resulting from Rasmussen's encephalitis, some patients showed seizure improvement following adalimumab administration, an anti-TNF-a therapy. In this study, patients had over a 50% decrease in seizure frequency and shown an improvement in their functional deficit. Further studies are necessary to confirm the results of the efficacy of adalimumab and its further therapeutic implications in epilepsy (146).

### Anti-inflammatory Cytokines in Epilepsy

#### IL-1Ra

IL-1Ra, the antagonist of IL-1 receptor type 1, limits IL-1B-mediated actions through the inhibition of IL-1B's biological activities and its receptor binding. IL-1Ra is induced in response to seizures, and is indicated to exert neuroprotective and anticonvulsant effects. Increased levels of IL-1Ra is observed after episodes of seizures. Its elevation after generalized seizures is higher than its increase after complex partial seizures, indicating its reflection on the seizure severity (21). In a study on neonatal seizures, IL-1Ra was continuously inactivated with significantly lower concentration in seizure group within 72 h of seizure attack. It is hypothesized that this lack of consistent IL-1Ra induction in response to epileptogenic environment may be characteristic of neonatal seizures, making the neonatal period more vulnerable to seizures (22).

#### IL-10

IL-10 plays an important regulatory, anti-inflammatory role, counteracting various pro-inflammatory processes during infection as well as in autoimmune disorders. The anti-inflammatory effects of IL-10 is mediated through the deactivation of macrophages, which in turn decreases the production of pro-inflammatory cytokine production by T-cells.

Although increased levels of pro-inflammatory cytokines were primarily found in patients with epilepsy, significant elevations of CSF IL-10 were also observed in epileptic patients (22). It has been hypothesized that the increase of IL-10 in CSF of epilepsy patients can be due to counteracting mechanisms to the pro-inflammatory stimuli. As an example, in neonatal seizures, IL-10 levels were elevated in plasma 48–72 h after seizure onset. This may indicate the enhanced protective role of IL-10 which has an anticonvulsive effect in neonatal seizure patients (22).

### Other Cytokines

#### EPO

Different cell types within the nervous system, including neurons, glial cells, endothelial cells, produces EPO, and expresses EPO-R (147). Several studies have demonstrated that EPO could enhance phagocytosis in polymorphonuclear cells and reduce the activation of macrophages, thus modulating the inflammatory process. EPO could play a protective role in neuronal survival after an epileptic seizure. A significant difference in EPO levels in the CSF has been observed between seizure groups and control subjects. Changes in the levels of EPO after generalized tonic-clonic seizures has been reported to positively correlate to the duration and frequency of seizures (148).

#### Hs-CRP

High-sensitivity CRP (Hs-CRP) is a useful biomarker to detect chronic, subtle inflammation, which is not detected by conventional CRP values. It is significantly higher in the daily generalized motor seizures than in either intermittent seizures or control (142).

#### CCL2

Chemokines, expressed in microglia, astrocytes, and endothelial cells, plays a role in the guidance of inflammatory mediators toward the source of inflammation and in the activation of leukocytes (149). CCL2 is one of the primary elevated inflammatory chemokines observed in patients with pharmacoresistent epilepsy. It is of particular interest after results from animal studies reveal its upregulated expression in addition to the enhancement of seizure frequency as a result of induced systemic inflammation. Inversely, exogenous administration of anti-CCL2 antibodies suppress LPS-mediated seizure enhancement in chronically epileptic animals. Although there are limited results from human studies, these observations may point to the significance of CCL2 in the molecular pathways that link peripheral inflammation with neuronal hyperexcitability (150).

In Table 2 we present the summary and characteristics of the above-mentioned factors.

Table 2Cytokines and their main role in epileptogenesis.

**Table:** 

	**IL-1B**	**IL-6**	**IL-8**	**TNF-a**
Action	• Pro-inflammatory cytokine • Elevated under certain pathological states (active seizure, hypoxic injury, infection)	• Pro-inflammatory cytokine • Involved in the acute phase of the immune response	• Pro-inflammatory cytokine promote neuronal growth after injury • Stimulates the production of nerve growth factor	• Pro-inflammatory cytokine
Generalized tonic-clonic seizures	• No changes in plasma levels, no significant differences • CSF IL-1B levels show an increase after seizure with a significant positive correlation with the duration and frequency of seizures	• Levels strongly increased after single and recurrent GTCS • IL-6 significantly higher in the daily generalized motor seizures than in either intermittent seizures or control	• Associated with seizure severity	• No significant differences
Partial seizures	• No changes in plasma levels	• Levels increased in prolonged partial seizures but to a lesser extent than in GTCS	• Elevated levels in serum	
Neonatal seizures	–	–	• Significantly increased within 24 h; remained increase after 48–72 h	
Mesial temporal lobe epilepsy	• Increased level in brain tissue	• Increase frequency of CD4+ T-lymphocytes expressing IL-6 • IL-6 increased in serum	• Associated with seizure severity	• Decrease frequency of CD8+ T-lymphocytes expressing TNF-a in mTLE patients
Febrile seizures	• No significant differences in CSF and serum	• No significant serum elevation		
References	(22, 127, 134–139)	(140–144)	(22, 136, 145)	(133, 142, 146)

**Table:** 

	**IL-1Ra**	**IL-10**	**EPO**	**CRP**	**CCL2**
Action	• Anti-inflammatory cytokine • Limits IL-1B-mediated pro-inflammatory actions through the inhibition IL-1B's biological activities and receptor binding • Neuroprotective and anticonvulsant effects	• Anti-inflammatory cytokine • Suppression of pro-inflammatory cytokine production	• Enhance phagocytosis in polymorphonuclear cells • Reduce the activation of macrophages • Possible protective role in neuronal survival after an epileptic seizure	• Biomarker to detect chronic, subtle inflammation	• Guide inflammatory mediators toward the source of inflammation • Activation of leukocytes
Generalized tonic-clonic seizures	• Levels increased after seizure, higher in generalized seizure than after complex partial seizures	• Increased level in CSF	• CSF EPO levels show an increase after seizure with a significant positive correlation with the duration and frequency of seizures	• High-sensitivity CRP (Hs-CRP), IL-6 significantly higher in the daily generalized motor seizures than in either intermittent seizures or control	• Elevated in patients with pharmacoresistent epilepsy
Partial seizures	• Levels increased after seizure	• Increased level in CSF			
Neonatal seizure (hypoxic-ischemic encephalopathy-induced seizure)	• Levels increased within 24 h; rapid decreased after 48–72 h	• Increased within 24 h; remained increase after 48–72 h			
References	(21, 22)	(22)	(147, 148)	(142)	(149, 150)

**Figure 2:**
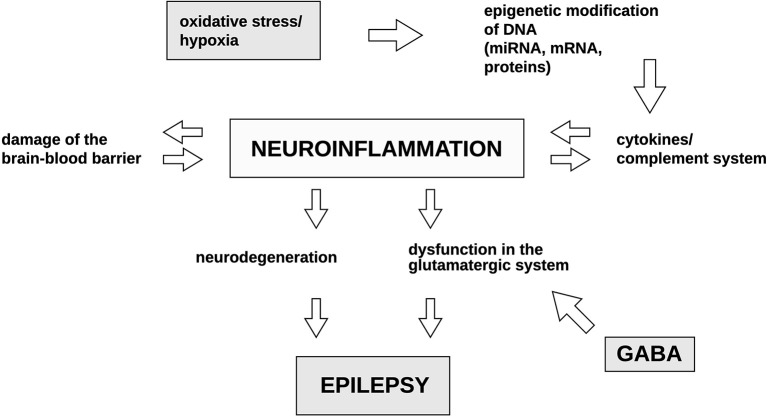
Potential mechanisms occurring during epileptogenesis and their correlations with one another. Both oxidative stress and hypoxia have been previously reported to induce epigenetic modifications of DNA. This may result in the activation of cytokines and the complement system. Consequently, the resulting neuroinflammatory processes may in turn induce the production of cytokines and the elements of the complement system in a feedback loop. Neuroinflammation is the primary factor which leads to BBB destruction, neurodegeneration and the dysfunction in the glutamatergic system resulting in the dysregulation of GABA synthesis. Culmination of the above-mentioned mechanisms result in epilepsy. GABA, gamma-Aminobutyric acid. Illustration by Paulina Szuba.

Figure 2 summarizes the discussed pathways which could potentially lead to the development of epilepsy.

## Proteins and Amino Acids Role in Epilepsy

### Aspartate, Glycine, Glutamate, and NMDA Receptor

The role of aspartate and its N-methyl-D-aspartate receptor (NMDA-R) has been widely discussed in the previous decade. A clear connection exists between this amino acid and epileptogenesis, though the exact mechanism is still debatable. NMDA-Rs are ionic glutamate receptors. Several characteristic attributes of NMDA-Rs include co-agonist activation, extracellular Mg^2+^-induced voltage-dependent blockade, elevated permeability to Ca^2+^, and slow gating and deactivation kinetics (151).

Aspartate and glutamate can both activate NDMA-R, with glutamate being the one with a stronger stimulation potential. Glycine is a required co-agonist. However, it can be exchanged for stronger binding D-serine (152). Ronne-Engström et al. presented the changes of amino acids level in extracellular fluid (ECF) taken by microdialysis from the epileptogenic brain regions of patients suffering from epilepsy. There was the greatest increase in the aspartate level, and the levels of glutamate, serine, and glycine also increased significantly (153). This indicated the possible role of NDMA-R in epileptogenesis.

There are different mutations concerning NMDA-R subunits. GluN1 subunit is encoded by GRIN1 gene at chromosome 9q34.3, with twelve mutations previously described (154, 155). GluN2A is encoded by GRIN2A gene at chromosome 16p13.2, which is a genome hotspot, and very susceptible to mutation, eighty-two of which were previously described (155, 156). These mutations are also suspected to be strongly connected with epileptogenesis. GRIN2B, encoding the CluN2B subunit is located on chromosome 12p13.1, with thirteen mutations connected with epileptogenesis (155, 157). One epileptogenic mutation is also known in the GRIND2D gene on chromosome 19q13.3, encoding the GluN2D subunit (155, 158).

Different mutations present with different disease phenotypes. GRIN2A mutation is commonly associated with childhood epilepsy syndromes within epilepsy-aphasia spectrum, such as benign epilepsy with centrotemporal spikes (BECTS), Landau-Kleffner syndrome (LKS), and epileptic encephalopathy with continuous-spike-and-waves-during-slow-wave-sleep (CSWSS) (159, 160), whereas GRIN1, GRIN2B and GRIN2D mutations present with developmental delay and more severe phenotypes of epilepsy (161, 162). This is connected with the embryonic expression of subunits encoded by these genes and the fact that GluN1 is a subunit required for proper functioning of NDMA-Rs throughout the brain (163). However, GRIN2A can also lead to more severe phenotypes, such as early-onset epileptic encephalopathy. Among relatives, the genetic penetrance may vary and thus not every member will be affected by epilepsy (155).

Studies have indicated the inflammatory background of NMDA-R-associated epilepsy. IL-1β and HMGB1 use interleukin 1 receptor type I (IL-1R1) and Toll-like receptor 4 (TLR4), respectively, to activate Src kinases-dependent NR2B phosphorylation and to enhance NMDA-mediated Ca^2+^ influx (164, 165). Furthermore, HMGB1 uses physical, non-receptor interaction with presynaptic NMDA receptors, resulting in the release of Ca^2+^-dependent glutamate. This mediates inflammatory cell loss and epileptogenesis by the excitability of CA1 neurons via reduced NMDA-induced outward current (166). NMDA-dependent long-term synaptic depression in the hippocampus is also connected with the activation of JAK/STAT pathway by IL-6, which has a significantly higher occurrence of epileptogenic neuronal damage (167). Additionally, the exposure to lipopolysaccharides in early childhood correlates with a further poor developmental outcome due to chronic changes to NDMA-R and its units' expression in hippocampus and cortex (168, 169).

Martell et al. showed the correlation between the activation of NMDA receptors and voltage-dependent intrinsic oscillations in intracellular whole-cell patch clamp recordings of neocortical pyramidal neurons, with a simultaneous instability of the neuronal system, presented by the whole-cell I-V curve and the lower frequencies in resonance. This suggests the role of NMDA-R in both producing low-frequency oscillations and in promoting cell responsiveness to lower frequencies, which could make these neurons more prone to epileptogenesis (170).

In a recent study involving post-traumatic epilepsy (PTE)—kindled rat models and a small group of patients with temporal lobe refractory epilepsy, Liu et al. (171) shows a significant decrease in microfilament heavy chain level in the epileptic brain tissue, which is a reflection of axonal impairment. He also observed an elevated level of amyloid precursor protein (APP), but its contributions toward epileptogenesis remain unclear. On one hand, reduced level of APP is connected with increased susceptibility to seizure, as reported by Steinbach et al. (172). However, the elevated level of APP is also connected to hyperexcitability (173). What was observed by Liu et al. was the ability of NMDA-R antagonists to both counteract the accumulation of APP and reverse previous accumulations, while NMDA-R activation can lead to the blockage of axonal transport which is crucial for maintaining physiological neuronal function. The hypothesis is that the process is controlled by the upregulated activity of Cdk5 and GSK-3β (neurofilament kinases), in which elevated levels were observed in brain tissues of both human and rat models. Both kinases use different pathways to slow down the axonal transport. GSK-3β downregulates kinesin-based motility (174) and increases neurofilament bundling (175), while Cdk5 uses the phosphorylation of NFH side arms (176), and the pathway via Lis1/Ndel1-dependent regulator in conditions of stress (177).

The potential clinical impact of these findings was tested on a rat model with kainic-induced SE, relating to limbic system protection by NDMA-R inhibitors. Hippocampal and limbic system damage has been considered as potential starting points for the later development of epilepsy among children with febrile seizures (178). Studies have also indicated that the lesions in the limbic system underlie the predisposition to febrile seizures (179) or are secondary to early epileptic signs (180, 181).

Therefore, the clinical implication of limbic system damage and its role in neuroprotection remains equivocal. The inhibition of NMDA-R by dizocilpine after SE showed processes of neuroprotection in most affected limbic system regions, except for the hilus of the dentate gyrus and the substantia nigra pars reticulata. Although the hilus was susceptible to damage during SE, it was not progressive in the NDMA-R inhibitor group. Thus, dizocilpine was suggested to have a potentially protective role. A reduced level of fragmented DNA and histological apoptosis markers suggests that the inhibition of NDMA-R could prevent neuronal apoptosis. The prevention of the loss of dentate granule cells is of clinical importance as the resulting hyperexcitability in damaged regions may ultimately lead to the development of epilepsy. However, the neuropathological indication of neuronal protection did not correlate with the clinical prevention of spontaneous recurrent seizures (SRS) (182). Additionally, a study on ketamine, another NDMA-R antagonist, on pilocarpine-induced SE rat model reproduced similar results to those from the kainic model concerning the development of SRS (183). On the other hand, the limitation of the dizocilpine study was the injection of only a single dose of NMDA-R inhibitor, which cannot exclude the fact that, with repetitive inhibition, the neuropathological protection could be followed by clinical improvement (182).

The development of proper treatment based on NDMA-R inhibition is still an ongoing process, with its first data reported mostly through case studies (155). In the case of a 6-year-old child with GRIN2A mutation and early-onset epileptic encephalopathy, non-responsive to conventional methods of treatment, good response to memantine (159), an FDA-approved drug used clinically for the treatment of Alzheimer's disease, was observed. In rat models, memantine was observed to significantly lower the reduction of NFH by decreasing Cdk5 and GSK-3β, showing probable mechanisms of its protective role. GLuN1 and GLuN2B inhibitor, ifenprodil, showed similar, promising results (171). A number of experiments of *in vitro* electrophysiological models included different NMDA-R inhibitors, such as ketamine, magnesium, dextromethorphan, dextrorphan, amantadine, and TCN-201 (159). TCN-201 in rat models significantly reduced the number of epileptiform events (184). The most commonly used inhibitor, memantine, occurs as a stronger, and safer NMDA-R inhibitor than amantadine (185). Ketamine has lower potency and is therefore less effective than memantine. An analysis of the potential of a selective GluN2B negative allosteric modulator, radiprodil, was proven to be more effective than other NDMA-R inhibitors in some variations of gene mutations (186).

NDMA-R inhibitors are not always equally effective when used in monotherapy. In a case study of two children with GluN2D V667I mutation, one remained refractory to the monotherapy of memantine, midazolam, pentobarbital, ketamine or magnesium, while polytherapy with ketamine and magnesium i.v. proved to be beneficial. In another case, in which the patient was found to be refractory to memantine and polytherapy of memantine, sulthiame, and lamotrigine improved his condition. This indicates the possible usage of NDMA-R antagonists in polytherapy along with conventional anticonvulsant (158).

### The Role of Glutamine Synthetase

Glutamine synthetase (GS) is an enzyme characteristic for astrocytes. It has a leading role in the glutamine-glutamate-ammonia cycle. Glutamine is involved in many biological processes including the Krebs' cycle and is a precursor to the neurotransmitters gamma-aminobutyric acid (GABA) and glutamate (187, 188). It connects processes of cell metabolism, the detoxification of ammonia, glutamate, and the neurotransmitter pool role (189, 190).

Physiologically, glutamate released from synapses is converted by GS into non-toxic glutamine due to its uptake by glial cells, mostly via the excitatory amino acid transporter 2 (EAAT2), and then transported back to neurons, repeating the cycle (191, 192). GS is expressed on glial cells, where it is responsible for 80% of glutamate transport (193, 194). The cycle of glutamate and glutamine is also affected by the malfunction of the EAAT2 (195, 196), phosphate-activated glutaminase (197) and the vesicular glutamate transporter 1 (VGLUT1) (198).

Chronically elevated levels of extracellular glutamate lead to increased excitotoxicity (199–201), as observed in various neuropsychiatric disorders (202), including refractory epilepsy. The studies on GS after SE in animal models are equivocal. In kainate models, in the latent phase GS expression was higher, while it decreased in the chronic phase (203, 204). In the pilocarpine model, however, GS in the chronic phase appeared redistributed rather than downregulated (205).

Mesiotemporal sclerosis (MTS) is characterized by region-specific neuronal loss (206–208), reactive alterations in astrocytes, gliosis, and mossy fiber sprouting (209, 210). Astrogliosis, a characteristic feature for MTS, presents with an upregulation of the intermediate filament marker, glial fibrillary acidic protein (GFAP) (211–214). This pathomechanism has been observed in TLE, a type of refractory epilepsy (215).

In TLE patients and in rodent epilepsy models, regions of the hippocampus affected by cell death shows downregulated GS, leading to an increase in extracellular glutamate concentration, ultimately resulting in neural hyperexcitability, excitotoxicity, and neurodegeneration in epilepsy (205, 216). Using magnetic resonance spectroscopy, it was observed that what underlies this downregulation is the disruption of glutamate-glutamine cycle. Not only was the glutamate level increased and the glutamine level decreased, the process of cycling was slower (217). The glutamate receptor subunits GluR1 and GluR2 in the hippocampus appeared to be upregulated. In two studies of resected tissue obtained from subjects affected by TLE, however, no changes in glutamate transporter expression were found (215, 218).

In brain microdialysis studies, it was observed that the increase in glutamate level occurred seconds before a seizure started and the peak was observed for no shorter than 15 min after the end of EEG recordings of the seizure (215, 219). Analogs of glutamate or glutamate itself can trigger seizures and its antagonists counteracts a seizure occurrence (220, 221). In addition, genetic deletion in GS or EAAT2 expression (222) or the injection of its inhibitor such as methionine sulfoximine led to spontaneous seizure in a rodent model (223–226). Currently, there are no known substance that would alleviate epileptogenesis caused by GS or EAAT2 disruption. The role of genetic engineering in treatment is yet to be described.

### P2X7 Receptor (P2X7R)

One of the most discussed receptors with a well-studied role in epileptogenesis is P2X7R, a cell surface-expressed, purinergic, ionotropic receptor for ATP, which is released in the event of neurotrauma (e.g., seizure). In rodent models, P2X7R, in contrast to other receptors from P2X family, is only expressed in the postnatal period, reflecting a correlation with CNS maturation and the development of purinergic signaling, required for proper development (227–229). Abnormalities in purinergic signaling lead to abruptions in neuronal migration and axonal outgrowth, disrupting proper synaptogenesis, and the development of microglia and astroglia (230, 231).

ATP release roots through both a physiological, activity-regulated manner and through neuronal and glial damage. All of P2X receptors bind to ATP (232). P2X7R requires a high amount of this ligand, a characteristic feature for pathological conditions (233). The activation of P2X7R is connected with immunological reactions of microglia and the release of caspase-1-dependent interleukin-1b (IL-1b), regulated by inflammasomes (234). This interleukin, in turn, promotes glutamatergic signaling and upregulates the activity of cycloxygenase-2 (COX2), nitric oxide synthase (NO synthase) and TNF-a, leading to increased excitability (125, 235). The expression of P2X7R on a molecular level seems to be regulated by the Specificity protein 1 (Sp1) transcription factor in neuro 2a cells (236) and posttranscriptional silencing by microRNAs. The latter was observed in mice model with induced status epilepticus (237).

TNF-a is primarily a product of macrophages and T-cells, existing at low levels in the physiological brain. It can be rapidly upregulated in pathological conditions by glial cells, neurons and the epithelium. The main pathways of TNF-a activity include NF-kB binding leading to cell death (238) and the activation of p38 mitogen-activated protein kinase resulting in cell survival (239). Studies on the P2X7R agonist, 2′-3′-O-(benzoyl-benzoyl) ATP (BzATP), and its antagonist, oxidized ATP (OxATP), indicates a crucial role of TNF-a in the homeostatic balance between neuronal cell death and neuroprotection. The activation of P2X7R, followed by TNF-a activation, was shown to reduce glutamate-induced neuronal cell death (240, 241). The modulation of P2X7R by its agonists and antagonists in a rat pilocarpine epilepsy model indicated that the activation of P2X7R and its induction of TNF-a can lead to more evident neuronal damage within the hippocampus (242). However, in KASE model, P2X7R antagonist treatment was not associated with astroglial protection (243, 244).

The inhibition of P2X7R is also connected with the reduction of neutrophil infiltration after SE via Monocyte Chemoattractant Protein 1 (MCP-1) (245–247). Immunoreactivity is detected in microglia and further regulates the activity of Macrophage Inflammatory Protein 2 (MIP-2), leading to neuronal damage (248). In addition, P2X7R modulates glutamate and GABA release in the hippocampus (233, 249–252), lowering the intracellular potassium level and depolarizes sodium and calcium entry (253). It is possible for P2X7R to modulate the activity of neurons by PanX1, a membrane channel opened by P2X7R, which modulates neuronal cell death and neuronal activity (254).

P2X7R is upregulated in seizures within the hippocampus and the cortex in mouse models. This upregulation in HI seizures models is prolonged, leaving the brain susceptible to further epileptiform events and epilepsy development (255). This would lead to a rapid or an enhanced release of pro-inflammatory cytokines such as IL-1b, resulting in a prolonged neuroinflammatory response and further injury (256, 257) and a disruption of cognitive and hippocampal function of brain regulated by IL-1b, which seem to be affected in HI seizures in rodent model (255).

In various clinical studies, the injection of P2X7R antagonists such as A-438079, JNJ-47965567, Brilliant Blue G and JNJ-42253432 lead to reduced seizure intensity. It also limits the immunological reaction via caspase-activation and neuroinflammatory genes transcription (232). The antagonists proved helpful in different neurological abnormalities, such as Alzheimer's disease, traumatic brain injury and Parkinson's disease (258–262). They were also studied in non-neurological conditions and appeared safe and well-tolerated, although they showed no efficacy in those diseases (263–265).

A-438079 injection proved effective in kainic rat models of status epilepticus. The neuroprotective outcome of A-438079 was also observed in global hypoxia invoked in rats, a model for neonatal hypoxic-ischemic (HI) seizures. However, the effect on post-hypoxia seizure was limited due to the short-term study duration. A high dose of the antagonist did not present comparable results, indicating a short duration of action and a narrow therapeutic window (251). In another study of a KASE epilepsy model, A-438079 combined with lorazepam caused seizures cessation during status epilepticus. However, it is unclear whether the drugs had a crucial role (266). Comparable results in HI seizures mice model was also observed for JNJ-47965567, with a similar clinical limitation for its usage (232). None of the antagonists presented full cessation of seizures, indicating that seizures can be triggered by a different neurotransmitter rather than by ATP (267, 268). JNJ-42253432 led to less severe phenotype of epileptiform activities though failed to suppress SRS (243).

Brilliant Blue G (BBG), a selective P2X7R antagonists reducing Ca^2+^ influx in neuronal cells, which in turn increases glutamate transporter 1 (GLT-1)/Glutamate aspartate Transporter (GLAST) mRNA stability, reducing glutamate release. This leads to the recovery of astrocytic GLT-1/GLAST function and consequential higher glutamate reuptake (269). This is crucial for the prevention of excitotoxicity. In rat models, BBG administration helped in PTZ-induced kindling animals to improve cognitive functions, such as learning and memory, which can be a clinical sign of reduced hippocampal injury and cell death (270). However, BBG had a non-satisfactory anticonvulsive effect in 6 Hz electroshock-induced mice model, not affecting the seizure threshold (271).

### Aquaporin 4 and Its Role in Neuroexcitation

Aquaporin 4 (AQP4) is a protein from the aquaporin family of hydrophobic membrane channels, serving as a water channel in accordance to the osmotic gradient (272–275).

AQP4 is expressed by glial cells in the brain and the spinal cord, mainly within points of contact between astroglial end-feet and blood vessels and astrocyte membranes ensheathing the glutamatergic synapses (276–278). In mice models, AQP4 deficiency has been connected to prolonged seizures along with deficit extracellular K^+^ clearance. This has been explained through the role of water and ion homeostasis in blocking hyperexcitability. Accordingly, the expression of AQP4 has been reduced in the perivascular membrane within the epileptically-altered sclerotic regions of the hippocampus (272). Moreover, AQP4 immediately decreases its immunoreactivity post-SE in kainic-induced epileptic mice models, which correlates with the prolonged seizures observed (279, 280). However, it is unclear whether this is due to the initial change during SE or that SRS trigger recurrent changes. The changes were observed mainly in stratum lacunosum moleculare, the molecular layer and the dentate gyrus, affecting the fine processes of astrocytes as well as its end-feet (272, 280, 281). Immunoreactivity diffused to a greater extent in neurophils, especially in the areas of dysmorphic neurons. In a compensatory manner, AQP4 mRNA levels are increased (272, 282). The exact mechanism of this reaction is unclear (280, 283). It is probable, in accordance to the mathematical modeling of the AQP4-deficiency model of water and ionic (potassium) transport in brain ECF, that post-neuroexcitation changes in rate and extent of alterations in extracellular space volume affect changed K^+^ dynamics and what is more, also on astrocyte water permeability. It may also have an influence on long-range K^+^ buffering and gap junction coupling (283). The other theory suggests that the cause and the result are the opposite: diffuse immunoreactivity in the piriform cortex and the hippocampus with an expression mostly observed at end-feet astrocytes, after SE results in areas lacking AQP4 in piriform cortex. The role of mislocalization of APQ4 with the reduction of channel in adluminal end-foot membranes rather than in the abluminal ones, that stable level is underlined in some papers, with the results of testing suggesting no changes in expression, but rather in the localization of AQP4 in subjects with epileptic seizures (284, 285). Both lowered expression and incorrect localization on end-foot membranes can lead an alteration in homeostasis. Additionally, AQP4 is described as a factor influencing synaptic plasticity by neurotrophin mediation, especially neurotrophin BDNF, leading to long-term potentiation, depression and location-specific object memory in mice models (286–290).

Glial fibrillary acidic protein (GFAP) is another astrocyte marker, characterized by its intermediate filaments. In kainate-induced SE, the levels of GFAP were visibly elevated in all areas of the hippocampus excluding the stratum sadiatum and stratum lacunosum moleculare. Immediately after SE, no changes in GFAP protein expression were observed but a trend toward increased protein was observed later post-SE, while GFAP mRNA anteceded the increase in GFAP levels (291, 292). It led to further sclerotization of the hippocampus, a phenomenon characteristic for further development of TLE. This is another indication of protein markers of astrocytes playing a role as a marker for the development epilepsy after an epileptiform event (272).

The theory of an inflammatory cause of epilepsy has not been reflected in the possible role of AQP4 in epileptogenesis. In mice models, AQP4 deficiency has an alleviating effect on experimental autoimmune encephalomyelitis as well as on inflammation after intracerebral lypopolysacharydes (LPS) administration (293–295). AQP4 stimulates AQP4-dependent cell to swell and promotes cytokine release. It also activates astrocyte Ca2+ signaling via TRPV4 as a reaction to an osmotic stimuli (284).

The therapeutic possibilities are currently limited. In several small studies, substances such as tetraethylammonium (TEA+), azetazolamide, carbonic anhydrase inhibitors, bumetanide, and its analog AqB013 and others may have the potential to inhibit AQP4, but the results remain inconclusive (296–302). Antiepileptic drugs, such as zonisamide, lamotrigine, phenytoin and topiramate, were observed as AQP4 inhibitors. The safety level of various substances with inhibiting capacities, including NSC168597, NSC164914, and NSC670229, is uncertain (303). A promising molecule is TGN-020, a structurally similar substance to carbonic anhydrase and antiepileptic drugs. Up to this point, its peritoneal injection was shown to reduce ischemic cerebral edema and infarct volume in a rat model of ischemic stroke, without any studies on its potential role in epilepsy treatment (303). Similar results have shown the effects of IMD-0354, an inhibitor of both kinase IKKβ and AQP4, in lowering the intracranial pressure in mice models after acute water intoxication and as a form of pro-drug (a phenol phosphate) to reduce brain edema, improving the neurological state of mice after an ischemic stroke (304). The studies conducted on different models are also prone to an assessment distortion due to potential factors affecting or mimicking AQP4's role and the need for blood-brain barrier penetration of potential inhibitors (284).

### Matrix Metalloproteinase-9 and Epilepsy

Matrix metalloproteinases (MMPs) are zinc-dependent endopeptidases which play a role in regulating the cell-matrix composition. They are produced by neurons and, to a lesser extent, by glial cells. They play a crucial role in prenatal (in embryogenesis and morphogenesis) and postnatal development (in remodeling of tissues). They could potentially play an important role in the pathomechanism of neurodegenerative disorders (such as Alzheimer's disease), ischemia, neurotrauma, neoplasms (305–308), inflammation (309) and epilepsy (310–314). In pathological conditions, the stimulation of MMPs is upregulated by cytokines from immune cells and glia (315–317).

MMP-9, a gelatinase, is activated extracellularly from inactive zymogen. Matrix metalloproteinases (TIMPs), especially TIMP-1, have a controlling role over MMP-9. MMP-9 can influence cerebral epithelium via the proteolysis of type IV collagen. To a lesser degree, it degrades other types of collagen (V and XI), laminin and aggrecan core protein. It is also involved in learning (318, 319) and in neuronal plasticity. It serves an important role in controlling the extracellular matrix protein composition (320, 321) through the proteolysis of molecules responsible for signaling and adhesion, growth factors (321–324) and receptors for neurotransmitters (321, 325). The changes in synapses and its structures facilitates synaptic transmission and, consequentially, the excitability of neurons (312, 318). It has also been reported to contribute to neuroinflammation and to neuronal apoptosis in epilepsy in animal models.

Synaptic transmission is also influenced via MMP-9 by NMDA and AMPA glutamate receptors, reducing its efficacy after multiple seizures, as observed in 4-aminopyridine (4-AP) induced epilepsy model (325–328).

There are various possible pathways of MMP-9 activation. During an epileptic activity, the activation of MMP-9 depends on Ca2+ entry (329). However, the prolonged synthesis and accumulation of the precursor form of MMP-9, resulting in increased functional MMP-9, is unlikely to depend on the immediate increases in neuronal Ca2+ levels. During kainine-induced seizures, Ser-proteases such as tPA/plasmin and thrombin stimulate the release of MMP-9 (330, 331). The active form of MMP-9 can be quickly transformed from the constitutive pool of zymogen (312). Further synthesis can be observed due to neuronal intermediate early genes activity (332) or to transcription activation in neurons, stimulated by pro-inflammatory cytokines such as IL-1b and TNF-a, released from glia (333–336). These cytokines may use MAPK/Erk pathway to activate transcription (321, 336). The inhibition of MMP-9 in microglial cells after LPS stimulation decreases the level of pro-inflammatory cytokines such as IL-1b and IL-6 and inhibits the transcription of iNOS (inducible nitric oxide synthase) (337), modifying the pro-inflammatory activity of MMP-9.

Several studies suggest that the CNS attempts to reduce alterations in neuronal excitability through neuroplasticity (338–340). However, no epilepsy model presents signs of neuroplasticity, such as aberrant synaptogenesis or axonal sprouting (341) or structural effects on dendritic spine density (342, 343), leaving this hypothesis unclear.

In pathological conditions in which MMPs are more stimulated to activation, MMP-9 activity leads to the disruption of BBB. The BBB leakage results in further immunological reaction and immune cells' recruitment and migration. This can contribute to a worsening of the state of the patient, causing brain edema, hemorrhage or further spreading of infarct (344–348). For instance, in rat models, the leakage led to epileptiform activity with a positive correlation between MMP-9 levels and seizure frequency (349, 350).

In rat models after kainate seizures, MMP-9 activity and MMP-9 mRNA levels were significantly increased exclusively in the hippocampal dentate gyrus, correlating with the changes in the hippocampal dendritic architecture. This could be connected to synaptic abnormalities, such as the quantity of synapses and dendrites and to dysregulated synaptic transmission (311, 312). MMP-9 knockout mice were less sensitive to pentylenetetrazol (PTZ) kindling-induced epilepsy, with a simultaneous decrease of mossy fiber synaptogenesis (313), while MMP-9 overexpression results in increased dendritic spine proliferation and the misposition of synaptogenesis in the hippocampus. There is a positive correlation between MMP-9 level and seizure duration in acute encephalopathic patients. In patients affected by viral infections, higher MMP-9/TIMP-1 ratio which was measured after prolonged febrile seizures is indicated to be connected with dysfunctional BBB (351) and an increase susceptibility to febrile seizures or encephalopathy (253). On the other hand, higher MMP-9 levels in the CSF is observed in these patients with bacterial infections of the CNS who present with neurological complications, such as secondary epilepsy (352). In patients with systemic lupus erythematosus, patients with higher levels of MMP-9 were more prone to seizure activity and other neuropsychiatric symptoms in the course of their disease (353). Whether or not MMP-9 plays a significant role in seizure-induced neuroapoptosis is a question which necessitates further research.

Excitotoxicity leading to neuronal and hippocampal apoptosis in conjunction with high MMP-9 activity was observed in kainate-induced epilepsy models (310, 354). In pilocarpine models of epilepsy, the same phenomenon was also observed. The apoptosis was connected to signals of neuronal cell survival, mediated by integrin, and interrupted by MMP-9, after pilocarpine-induced status epilepticus (355).

The homeostatic balance in MMP-9 levels can also play a protective role. Its protective homeostatic plasticity involves extracellular substrates, including integrins (321, 324, 356), cadherins (357) and b-dystroglycan (322, 323, 358), which helps to control dendritic spinal shape and induce its reversible loss by b-dystroglycan or ICAM-5, consequentially affecting the entire synapse (313, 358, 359). This effect depends on MMP-9 mRNA activity-dependent dendritic transport, enhanced in the kainate epilepsy model (311). It is vital in obtaining reduced neuronal excitability and thus, the optimal conditions for recovery (360). In mice models, lower levels of MMP-9 led to reduced seizure-evoked pruning of dendritic spines, leading to decreased neuronal loss (313).

In models with 4-aminopyridine (4-AP) induced seizures and in Wistar Glaxo Rijswijk (WAG/Rjj) rats, no cell damage was observed. 4-AP models presented with generalized cortical seizures and WAG/Rjj rats presented with absence epilepsies, which typically generate spike and wave discharges after 4 months of age. MMP-9 and zymogen levels were increased in regions affected by seizure activity in these models (regions of the seizures' generalization within the cortex in the 4-AP model and the thalamus and cortical regions in WAG/Rjj during higher seizure activity). In WAG/Rjj, additionally, a diurnal peak was observed, which correlates with the sleep-wakefulness transition and the seizure activity. This indicates that cortical seizures promote the precursor and the active form of MMP-9. MMP-9's elevation could be an effect of elevated neuronal activity rather than that of neuronal death, as no apoptosis was observed in the WAG/Rij model of absence seizures and in the 4-AP model in the zones affected by seizure propagation (361). Additionally, higher levels of MMP-9 in WAG/Rjj rats treated with the anti-absence seizure drug ethosuximide (ETX) were reported. This is due to the suppression of the sleep-wake disturbances until ETX started to interfere with sleep pattern, which resulted in the downregulation of MMP-9 (362, 363).

The therapeutic potential of MMP-9 inhibitors remains inconclusive. In animal models, the MMP-9 inhibitor, S24994, has a protective role on the hippocampus in kainate-induced epilepsy. In kainate or picrotoxin models, it reduces dendritic spines after seizure activity (313). Monoclonal antibodies can also be beneficial and genetic engineering could provide further insights.

As in the case of AQP4, there are FDA-approved drugs with an inhibiting potential. Tetracyclines, statins, resveratrol, estrogen, and indomethacin are medications which have been observed to reduce MMP-9 levels. Tetracyclines (minocycline, doxycycline), via the prevention of BBB leakage, reduced CNS inflammation and size of infarction (364–368). Statins (atorvastatin, simvastatin, pravastatin) improved clinical outcome in acute coronary syndrome patients (369, 370). In animal models, atorvastatin and minocycline reduced seizure activity and inhibited neuroinflammation and neuronal apoptosis (371–374).

In Table 3 we present the summery and characteristics of above-mentioned proteins.

**Table 3:** Proteins and their role in epileptogenesis.

**Type of protein**	**Aminoacids involved in epileptogenesis**	**Activation or regulation**	**Effects**	**Possible therapeutic substances**	**References**
N-methyl-D-aspartate receptor (NMDAR)	Aspartate, glutamate, glycin, serin	↑	Producing low-frequency oscillation, promoting cell responsiveness to lower frequencies	Magnesium, dextromethorphan, dextrorphan, dizocilpine, memantine, ifenprodil, ketamine, amantadine, TCN-201, radiprodil	(152, 153, 158, 159, 171, 182, 185, 186)
Glutamine synthetase (GS)	Glutamine, glutamate	↓	Increased extracellular glutamate concentration, leading to hyperexcitability, excitotoxicity, neurodegeneration	-	(199–205) (215–218) (213–219)
P2X7 receptor (P2X7R)	Glutamate (via caspase-1-dependent interleukin-1b)	↑	Increased excitability, reduced glutamate-induced neuronal cell death	A-438079, JNJ-47965567, Brilliant Blue G, JNJ-42253432	(232, 243); (232, 258–266); (267–271)
Aquaporine 4 (AQP4)	–	↓	Hyperexcitability, prolonged seizures	Tetraethylammonium (TEA+), azetazolamide, carbonic anhydrase inhibitors, bumetanide, AqB013, antiepileptic drugs (zonisamide, lamotrigine, phenytoin, topiramate), TGN-020, IMD-0354,NSC168597, NSC164914, NSC670229	(276–290) (296–304)
Matrix metalloproteinase 9 (MMP-9)	Aspartate, glutamate, glycin, serin	↑	Disruption of blood-brain barrier, higher seizure frequency, increased susceptibility to febrile seizures	S24994, monoclonal antibodies, tetracyclines (minocycline, doxycycline), statins (atorvastatin, simvastatin, pravastatin), resveratrol, estrogen, indomethacin	(305–375)

## Conclusions

In this review, we summarize the current findings on the potential biomarkers of epilepsy. For the first time, we suggest that both processes of hypoxia and oxidative stress may lead to a neuroinflammatory state, ultimately resulting in epileptogenesis. Inflammatory factors may play an essential role in epilepsy. MiRNAs, regulated by epigenetic modifications, can be detected from biofluids. The diverse pathways and numerous molecules from recent investigations provide opportunities for further research regarding the diagnosis and treatment of epilepsy. The level of cytokines can be used to predict the disease severity and be useful in monitoring treatment efficacy. Medications targeting cytokines inhibitors can improve disease prognosis.

There is no consensus in which miRNA, protein or amino acid could serve as an ideal marker for epilepsy and further neuronal damage. Its connection to epilepsy is most likely through features connected with specific epileptiform events, rather than generally to epilepsy as a uniform disease. Each type of epilepsy presents with a different seizure phenotype, distinct behavioral changes, and further complications and comorbidities, suggesting the possibility of differences in the underlying etiology on a molecular level.

There are some common limitations among many studies on the molecular etiopathogenesis and development mechanisms in epileptiform events and epilepsy. First of all, only a few studies are performed on human cell lines. Even in these cases, the sample is not obtained from biopsy, but from fresh cadavers or during surgical treatment of neoplasms or epileptic lesions. Due to this collecting method, the sample obtained may have been altered and even damaged on the molecular level, leading to disturbances in studies results. Due to genetic modifications, the rodent model is becoming increasingly accurate in its resemblance to the conditions of human CNS but it could not serve as a relevant biological model. Secondly, the processes of inducing seizures can have a great influence on the behavior of neurons, glia cells, and their proteins. Thirdly, most studies presented results from a small sample size over a limited period of time. This can also lead to biased results and disturbances in their statistical analysis.

It is important to pay attention to the increasing number of molecules with a future therapeutic potential which are under investigation due to their influence on proteins and amino acids. There is also an open field for genetic engineering to enhance the power of established particles to regulate the excitability of brain cells. Nonetheless, we should remember that small rodent groups may not develop potential adverse reaction which may on the other hand be evident in human organisms. Because of this, FDA-approved drugs with modifying potentials can be the first step to novel therapy, based on protein, and amino acids activity in CNS. Extensive data exists regarding the molecular details of epileptogenesis. Although there are no conclusive answers, we can establish a starting point for further research on the therapeutic potential and clinical implications of proteins and amino acids reactions and collaboration in brain electric homeostasis. The role of finding novel markers of brain damage after post-epileptiform events is a possible grasping point for the prevention of complications and for new, targeted methods of treatment in the future.

## Author Contributions

DK was responsible for the Project administration, supervision, visualization, writing–review and editing. PI and WK were responsible for supervision, writing–review and editing. ZL, NL, SS, and AL were responsible for writing–review and editing.

### Conflict of Interest Statement

The authors declare that the research was conducted in the absence of any commercial or financial relationships that could be construed as a potential conflict of interest.
